# Forward Osmosis Technology and Its Application Progress

**DOI:** 10.3390/membranes16070220

**Published:** 2026-06-26

**Authors:** Bo Zhang, Ronggang Wang, Feng Wang

**Affiliations:** 1 Guizhou Branch, China Three Gorges Corporation, Guiyang 550081, China; zhang_bo17@ctg.com.cn (B.Z.); wang_feng17@ctg.com.cn (F.W.); 2 Guizhou Branch, China Three Gorges Renewables (Group) Co., Ltd., Guiyang 550081, China; 3 School of Mines, China University of Mining and Technology, Xuzhou 221116, China

**Keywords:** forward osmosis, membrane materials, draw solution, membrane fouling, lithium extraction

## Abstract

As a novel membrane treatment technology, forward osmosis (FO) has become a research hotspot in the field of membrane technology owing to its advantages such as low energy consumption and low pollution. Nevertheless, this technology still faces notable limitations, including lower water flux than reverse osmosis (RO), difficult regeneration of draw solutions, limited commercial membrane types, and unavoidable reverse solute flux, which restrict its large-scale industrial application. This paper reviews the characteristics of forward osmosis membranes, the classification of draw solutions, the characteristics of membrane fouling, as well as the applications and development trends of forward osmosis technology. Common FO membranes include cellulose acetate (CA) membranes, thin-film composite (TFC) membranes fabricated by interfacial polymerization, and aquaporin (AQP)-based biomimetic membranes. According to the types of draw solutes, draw solutions can be classified into gaseous solutions, organic compound solutions, inorganic compound solutions, magnetic nanoparticle-based draw solutions, polymer gel draw solutions, etc. Since FO is operated without external hydraulic pressure, it exhibits lighter membrane fouling compared with pressure-driven membrane separation technologies. FO membrane fouling can be mainly divided into four categories according to fouling types: inorganic fouling, organic fouling, colloidal fouling, and biofouling. FO technology has a wide range of applications and plays an important role in seawater desalination, pressure-retarded osmosis (PRO) power generation, wastewater treatment and reuse, and the energy field. Notably, the reconcentration and regeneration of draw solutions remain major energy and economic limitations restricting the large-scale deployment of FO. As a promising treatment technology, with continuous technological advances in membrane materials and draw solutions, FO will play a significant role in the energy field, especially in lithium extraction from geothermal water, promoting the iteration of forward osmosis technology from a “water treatment technology” to a “core technology for energy and resource recovery”.

## 1. Introduction

Water scarcity and the growing demand for efficient wastewater treatment and resource recovery have driven continuous innovation in membrane separation technologies. Conventional pressure-driven processes such as reverse osmosis (RO) are widely commercialized, but they face inherent limitations including high energy consumption, severe irreversible fouling, and poor adaptability to hypersaline feed streams. These constraints have created growing interest in low-pressure, mild-operation alternative separation platforms.

Forward osmosis relies on natural osmotic transport, a mechanism distinct from the hydraulic driving principle of reverse osmosis. Forward osmosis also employs a semi-permeable membrane, generally made of polymer materials, which allows the passage of water molecules but blocks the penetration of substances such as salts, carbohydrates, starch, and proteins [1]. Nevertheless, the full FO system still requires external energy for water pumping, solution circulation, feedwater pretreatment, and most importantly, the regeneration and reconcentration of draw solutions. Benefiting from its overall low energy consumption, mild membrane fouling and high water recovery rate, FO has attracted widespread attention worldwide as a promising energy-saving and emission-reduction membrane technology. Even so, FO has inherent drawbacks that cannot be ignored: its water flux is generally inferior to that of RO; the regeneration of draw solutes/agents usually brings extra energy and cost consumption; reverse solute flux remains a persistent challenge; and the variety of commercially available FO membranes is quite limited. These issues collectively hinder its further industrial promotion [2,3].

Despite extensive laboratory research and pilot demonstrations over the past two decades, FO technology still faces well-documented technical and economic barriers. Internal concentration polarization, reverse solute flux, membrane fouling, and high draw solution regeneration costs collectively limit its transition from bench-scale studies to large-scale commercial application. Most existing reviews focus heavily on material development and performance optimization, while few provide a systematic critical assessment of the gap between laboratory results and industrial viability, or a comprehensive techno-economic comparison with competing technologies.

Therefore, this review will systematically study the current status and latest progress of forward osmosis water treatment technology from the above aspects, and expand and explore its application fields.

## 2. Forward Osmosis Membranes

A standard FO application system comprises a complete set of functional units. The general workflow is as follows: raw feed water is firstly pretreated to eliminate particulate pollutants; thereafter, water permeates through the FO membrane driven by osmotic pressure and mixes with draw solution; the diluted draw solution is then reconcentrated via dedicated regeneration facilities and reused, while the produced water is collected for subsequent utilization.

**Figure 1:**
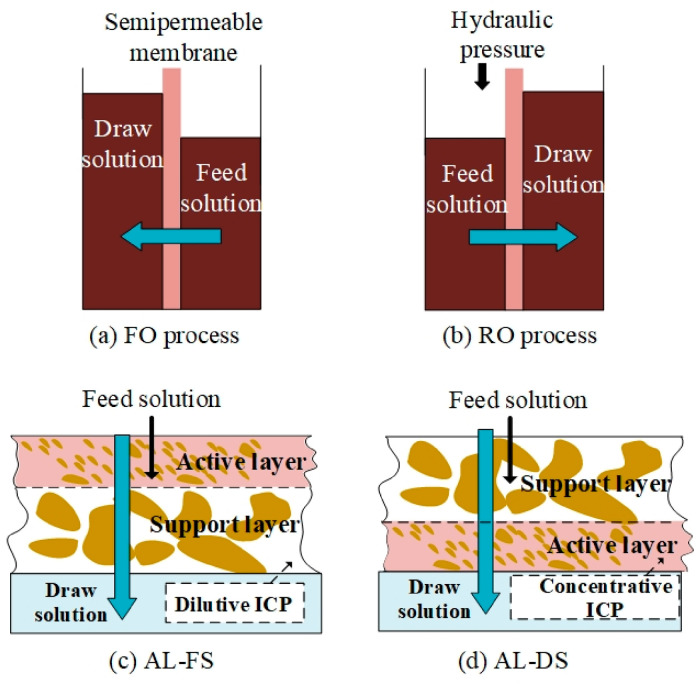
Comparison of Reverse Osmosis and Forward Osmosis Processes. AL-FS (Active layer facing feed solution), AL-DS (Active layer facing draw solution) (Adapted from Refs. [2,3]).

Both RO and FO membranes are composed of a dense selective skin layer and a porous support layer, but their structural design philosophies differ drastically (Figure 1). FO membranes are optimized for osmotic-driven separation without external pressure. They adopt a thinner, low-tortuosity porous support layer to minimize solute accumulation. Meanwhile, FO membranes require a highly dense active layer to suppress reverse solute flux. To sum up: RO membranes prioritize mechanical resistance for high pressure, while FO membranes focus on reducing concentration polarization and controlling solute leakage.

In conventional pressure-driven membrane processes, the feed solution permeates across the selective skin layer, and the permeate is collected on the other side; a thin dense skin layer delivers optimal water permeability and separation performance, while a relatively thick support layer provides sufficient mechanical strength and stability under high hydraulic pressure. Nevertheless, the thick porous support layer of conventional RO membranes tends to trigger obvious internal concentration polarization (ICP). This phenomenon is largely suppressed by the applied hydraulic pressure during normal RO operation; however, when traditional RO membranes are directly adopted for FO processes where no external pressure is supplied, severe ICP occurs and causes a sharp drop in water flux [4,5]. Multiple technical approaches are widely applied to relieve this issue, which can be divided into membrane structural modification and operational optimization.

In contrast to reverse osmosis, forward osmosis operates under negligible or zero hydraulic pressure, so there is no stringent requirement for the mechanical strength of FO membranes. Instead, FO membranes are demanded to possess high water permeability and low reverse salt flux. In response to these requirements, numerous research institutions worldwide have successively devoted efforts to the research and development of high-performance FO membranes. Hydration Technology Innovations (HTI) from the United States is one of the earliest commercial developers of FO membranes across the globe, and its commercialized FO membranes are highly representative and hold a dominant position in the global FO membrane market. In addition to HTI, other major international enterprises promoting commercial FO membranes include Oasys Water (USA), Aquaporin A/S (Denmark), Porifera (USA), and Modern Water (UK). Furthermore, a number of renowned universities and research institutes, such as Yale University, Nanyang Technological University, National University of Singapore, Ocean University of China, and Shanghai Advanced Research Institute of the Chinese Academy of Sciences, have made remarkable contributions to the fabrication and fundamental research of forward osmosis membranes [6].

The fabrication of forward osmosis (FO) membranes primarily relies on phase inversion, interfacial polymerization, and the vesicle rupture method. As the core material governing the FO separation process, an ideal FO membrane is expected to possess the following critical characteristics: (1) a selective active layer featuring high solute rejection, superior water permeability, and excellent hydrophilicity; (2) a porous support layer defined by thin thickness, high porosity, and robust mechanical strength; (3) strong interfacial adhesion between the active layer and support layer, coupled with satisfactory hydrophilicity and structural integrity; and (4) exceptional chemical stability, outstanding antifouling propensity, and broad tolerance to acidic, alkaline, and saline environments, thereby enabling versatile application scenarios. With the in-depth investigation into the structure-performance relationships of FO membranes, research efforts have predominantly focused on exploring design strategies and fabrication methodologies for high-performance FO membranes. The core research directions include developing novel membrane materials, optimizing the architectures of both the active layer and support layer, enhancing water flux, improving solute rejection efficiency, reinforcing antifouling properties, and augmenting mechanical robustness.

Commonly studied FO membranes encompass cellulose acetate membranes (CA), thin-film composite membranes (TFC) prepared via interfacial polymerization, and aquaporin (AQP)-based biomimetic membranes, among other representative materials.

### 2.1. Cellulose Acetate Membranes (CA)

Among all commercially available polymeric materials, cellulosic resins, especially cellulose acetate, stand out as the most extensively investigated materials for fabricating forward osmosis membranes. This is mainly attributed to their distinct merits, including low cost, superior hydrophilicity, facile membrane fabrication, and excellent chlorine resistance. Cellulose acetate is synthesized via the acylation of cellulose with acetic anhydride, and it features outstanding hydrophilicity, high water flux, favorable mechanical strength, chlorine resistance, oxidation resistance, and low fouling propensity, rendering it widely applied in forward osmosis processes. Among various cellulose acetate-based FO membranes, the unsupported double-skin-layer CA membrane features a unique structural design that can effectively alleviate concentration polarization and mitigate membrane fouling [7]. A double-skin-layer membrane is an advanced FO membrane structure with two dense selective active layers attached to both sides of the porous support layer, forming a sandwich structure: outer active layer–porous support–inner active layer. It is different from conventional single-skin membranes, which only have one active layer on the top surface of the support. Based on the solution diffusion model of single-skin-layer FO membranes, Tang et al. [8] established a mathematical model and successfully verified the advantages of double-skin-layer membranes in forward osmosis applications.

However, pristine cellulose acetate is prone to hydrolysis, exhibits poor biodegradation resistance, and has a narrow applicable pH range. Common cellulose derivatives include cellulose acetate (CA), carboxymethyl cellulose (CMC), hydroxypropyl methylcellulose (HPMC), hydroxyethyl cellulose (HEC), and ethyl cellulose (EC), each with distinct applications. For example, CA is widely used in FO/RO membranes, optical films, and cigarette filters; CMC serves as a thickener/stabilizer in food (yogurt, ice cream) and a binder in pharmaceutical tablets; HPMC is applied in controlled-release drug coatings and food emulsions; HEC acts as a rheology modifier in cosmetics and water-based paints; EC is used for enteric-coated formulations and moisture-resistant packaging films. The most widely employed CA-based membrane is the cellulose triacetate (CTA) membrane, which is an asymmetric polymeric FO membrane fabricated via the phase inversion method, and it is also one of the earliest commercial FO membranes applied in water treatment. Polymeric FO membranes prepared via phase inversion possess two distinctive characteristics: firstly, the active layer and the support layer are composed of the same material; secondly, the two layers are formed simultaneously during the fabrication process. Nevertheless, CTA membranes are relatively thick, and the skin layer and support layer are made of the same material with no distinct boundary between them, which hinders further structural and performance optimization of the membranes [9].

### 2.2. Thin-Film Composite Membranes (TFC)

In recent years, thin-film composite (TFC) membranes have emerged as the most intensively investigated category of FO materials, owing to their superior structural tunability and separation performance. The origins of interfacial polymerization can be traced back to the seminal work by Morgan in the 1960s [10], while a major breakthrough in the fabrication of TFC membranes was achieved by Cadotte in the 1970s [11,12,13]. Since then, interfacial polymerization has become the primary manufacturing technique for the fabrication of high-performance RO and NF membranes [14,15,16]. Structurally, TFC membranes are typically constructed via a two-step process: first, a porous support layer is fabricated using the phase-inversion method, followed by the interfacial polymerization of a thin, selective active layer on top of the support. The core advantage of TFC membranes lies in their decoupled structural optimization: the active layer and the support layer can be independently tailored to synergistically achieve high water permeability, excellent solute rejection, and robust antifouling properties. This dual-layer design strategy effectively addresses the trade-off between flux and rejection inherent in conventional single-polymer membranes, thereby enabling the precise tuning of FO membrane performance [17,18,19].

Currently, polyamide (PA)-based TFC membranes represent the most widely utilized configuration for FO applications. Yip et al. [20] pioneered the fabrication of PA-TFC FO membranes. In their work, a porous support layer was first prepared via phase inversion using polysulfone (PSf) as the polymer and N,N-dimethylformamide (DMF)/N-methyl-2-pyrrolidone (NMP) as the solvent. Subsequently, a PA active layer was formed on the PSf support through interfacial polymerization employing m-phenylenediamine (MPD) and trimesoyl chloride (TMC) as monomers. To further enhance performance, modifications to the support layer have been extensively explored. For instance, Wang et al. [21] prepared a high-performance support membrane by blending sulfonated polysulfone (SPSf) with hydrophobic polysulfone, which was then subjected to interfacial polymerization to fabricate the FO membrane. Regarding commercial membranes, Ren and McCutcheon et al. [22] systematically characterized the TFC membranes produced by Hydration Technology Innovations (HTI). Their results demonstrated that when the active layer was oriented facing the feed solution, using 1 mol/L sodium chloride as the draw solution and pure water as the feed solution, the membrane exhibited a high water flux of 22.9 L·m^−2^·h^−1^ accompanied by a salt flux of 6.4 g·m^−2^·h^−1^. This performance benchmark highlighted the great potential of TFC membranes in practical FO applications.

In recent years, electrospun nanofiber (ESNF) substrates have emerged as a revolutionary direction for optimizing TFC-FO membrane support layers, effectively overcoming the defects of traditional polysulfone (PSf) supports such as low porosity and severe internal concentration polarization (ICP) [23,24]. Electrospun nanofiber mats feature ultra-high porosity (75–85%), interconnected three-dimensional pore structures and adjustable thickness, which significantly reduce structural resistance and ICP, and further elevate water flux while maintaining high solute rejection. Current research mainly focuses on single-component nanofiber supports, dual-layer tiered nanofiber substrates and layer-by-layer (LbL) modified nanofiber supports [25,26,27]. Dual-layer nanofiber TFC membranes integrate the advantages of high flux and strong mechanical stability, and exhibit excellent anti-fouling performance during long-term cross-flow operation. Via polyelectrolyte deposition modification, PVDF-based electrospun nanofiber supports can gain enhanced hydrophilicity, with the optimized TFC membrane achieving a water permeability of 4.12 L m^−2^h^−1^ bar^−1^ and a low structural parameter of 221 µm, far outperforming mainstream commercial FO membranes. Nevertheless, the large-scale fabrication of electrospun nanofiber supports still faces challenges, including equipment cost and poor roll-to-roll processability, which restrict their industrial promotion.

### 2.3. Biomimetic Membranes

Biomimetic membranes are designed to emulate the excellent properties of biological membranes by mimicking natural structures, thereby achieving practical utility. The major challenge in fabricating biomimetic membranes lies in embedding aquaporins into the membrane matrix. Aquaporin (AQP)-based biomimetic membranes can achieve a water permeability as high as 6010 L/(h·MPa·m^2^) [28]. Aquaporin membranes represent a novel type of forward osmosis (FO) membrane that has emerged in recent years. Aquaporins are proteins that allow only water molecules to pass through while completely rejecting other solutes. Aquaporin membranes are fabricated by directly or indirectly incorporating aquaporins into an organic membrane matrix, resulting in biomimetic membranes with highly selective water permeability. Based on their structural characteristics, AQP-based biomimetic membranes are mainly classified into two types: those utilizing supported bilayer membranes as the AQP carrier, and those encapsulating AQP-containing vesicles [29]. The former enables high activity of the incorporated AQPs, while the latter offers enhanced mechanical strength and stability.

Compared with conventional commercial membranes, AQP-based biomimetic membranes exhibit significantly improved water flux and separation performance. However, the performance of currently developed AQP-based biomimetic FO membranes remains far below the theoretical ideal. This is primarily attributed to the susceptibility of AQP quantity and activity to fabrication conditions, as well as the variation in AQP activity under different environmental conditions during membrane preparation [30,31].

A comparison of the preparation methods and performance characteristics of different types of FO membranes is summarized in Table 1 [20,32,33,34,35,36,37]. Furthermore, as shown in Table 1, although certain fabrication methods have yielded AQP-based biomimetic membranes with relatively high water flux, the reverse salt flux of these membranes has not yet reached the desired low levels. Regarding AQP-based biomimetic membranes, several aspects warrant further improvement, including increasing AQP loading within the membrane, preventing AQP deactivation, addressing vesicle detachment issues, enhancing membrane mechanical properties and applicability, eliminating defects in the selective and support layers, and avoiding disruption of aquaporin functionality [29,38].

There is a notable gap between the laboratory performance and the practical scaled performance of AQP biomimetic membranes. In laboratory environments using pure water and stable operating conditions, AQP membranes deliver ultra-high water flux and ideal selectivity. Nevertheless, these advantages cannot be fully maintained in real applications. Long-term operation with complex raw water will gradually deactivate aquaporin proteins and impair membrane structural integrity, causing flux decline and increased reverse salt flux. In addition, the high cost of aquaporin materials and sophisticated manufacturing techniques severely restrict the large-scale commercialization of AQP membranes. Currently, this type of membrane still faces challenges in operational stability and economic efficiency when moving from laboratory research to industrial use.

Combined with the performance data in Table 2, the inherent trade-offs of three mainstream FO membranes are further analyzed. As the earliest commercialized FO material, CA/CTA membranes dominate low-cost scenarios owing to their low price and excellent chlorine resistance. However, their narrow applicable pH range and hydrolysis problem restrict their application in complex wastewater with strong acid or alkali. TFC membranes have become the most popular research object due to their high flux, high salt rejection and wide pH adaptability, but the polyamide selective layer is easily damaged by chlorine and susceptible to irreversible fouling, which increases the frequency of chemical cleaning. AQP biomimetic membranes possess the optimal water permeability and separation selectivity theoretically, yet the high preparation cost, unstable aquaporin activity and insufficient mechanical strength make them far from large-scale commercialization. At present, there is no single FO membrane that can simultaneously achieve high flux, low reverse salt flux, strong stability and low cost. Balancing these contradictory performances remains the core goal of FO membrane development.

As the core component of FO technology, the advancement in membrane fabrication and the reduction in membrane cost are undoubtedly critical drivers for the commercial application of FO technology. Although existing FO membranes have achieved considerable progress in water permeability and antifouling performance, two prominent problems remain unsolved. First, the types of commercial FO membranes are far less abundant than RO membranes, and most high-performance FO membranes are still in the laboratory research stage. Second, reducing reverse salt flux while maintaining high water flux is still a major technical difficulty for current membrane development. Therefore, future research and development will focus on FO membrane materials with high mechanical strength, good chemical stability, strong hydrophilicity, and low cost, as well as symmetric membranes that exhibit reduced internal concentration polarization.

## 3. Draw Solution

Selecting an appropriate draw solution is crucial for the development of the FO process. A suitable draw solution not only enhances the performance of the FO process but also reduces costs during the separation and recovery stage. If the cost and energy consumption associated with draw solution recovery become excessively high, the advantages of the FO process over other membrane processes (such as RO) will be significantly diminished. Draw solutions are high-osmotic-pressure media supplying the osmotic driving force for forward osmosis and are central to stable FO system operation. An ideal draw solution should possess the following characteristics: (1) high osmotic pressure; (2) low reverse permeation of the draw solute; (3) minimal concentration polarization; (4) safe presence in water; (5) no chemical reaction with the membrane material; and (6) recyclability and reusability [39,40]. Among these, osmotic pressure is particularly critical, as the osmotic pressure difference across the FO membrane serves as the driving force for the process. Therefore, the draw solution should exhibit high water solubility and osmotic pressure; the greater the osmotic pressure relative to that of the feed solution, the higher the water flux in FO.

Multiple mature technologies are available for the regeneration of the draw solution. The mainstream approaches cover thermal distillation, reverse osmosis, nanofiltration and membrane distillation. For gaseous and thermo-sensitive draw solutes, thermal decomposition and distillation are the primary choices. For inorganic and organic solutes, pressure-driven membrane processes are the dominant regeneration methods in practical projects. Although various types of draw solutions exist, the overarching goal of these studies remains unchanged: to enhance the efficiency of the FO process while minimizing the cost associated with draw solution recovery. Based on existing forms and working mechanisms, FO driving media are divided into two major categories: soluble draw solutes that form homogeneous draw solutions, and solid/colloidal draw agents. Conventional soluble draw solutes include gaseous substances, organic compounds and inorganic compounds. Differently, magnetic nanoparticles and polymer gels act as typical solid/colloidal draw agents rather than dissolved solutes. Unlike soluble solutes that dissolve completely in water to generate osmotic pressure, magnetic nanoparticles and polymer gels exist as solid particles or colloids. They function as draw agents without forming true homogeneous solutions.

### 3.1. Gaseous Draw Solutions

Some acidic or basic volatile gases exhibit high solubility in water and can generate considerable osmotic pressure, rendering them promising draw solutes. Notably, these gaseous draw solutes can be readily recovered via heating to realize cyclic reuse, which aligns with the requirement of low-cost regeneration for ideal draw solutions. Gaseous compounds, including ammonia(NH_3_), carbon dioxide (CO_2_), their mixtures and ammonium bicarbonate (NH_4_HCO_3_), are widely adopted as draw solutes in forward osmosis systems, each with distinct practical applications [41].

Ammonia solution is commonly used for concentrating high-salinity brines and treating industrial wastewater, while carbon dioxide aqueous solution features low toxicity and mild chemical properties, making it suitable for food-grade water purification and softening. The combined ammonia and carbon dioxide system shows strong adaptability to complex wastewater such as landfill leachate and mining effluent, and ammonium bicarbonate, formed by the reaction of ammonia and carbon dioxide, is preferably applied in facilities with abundant waste heat, like geothermal and thermal power plant water treatment systems. Ammonia-based solutions deliver high osmotic pressure and are easy to regenerate via thermal treatment, but they suffer from strong volatility, pipeline corrosion and potential secondary pollution. Carbon dioxide solution is low-cost, eco-friendly and barely corrosive, yet it provides limited driving force and cannot handle high-salinity water [42,43]. The mixed ammonia-carbon dioxide solution achieves a good balance between performance and stability, though its regeneration still requires heat input, and an improper ratio will increase reverse solute flux. Ammonium bicarbonate has low reverse solute flux, and it can be fully recycled by using low-grade heat to realize energy cascade utilization; however, it decomposes rapidly above 30 °C, restricting the operating temperature range and long-term operation stability. Notably, the economic and energy-saving merits of all these gaseous draw solutes depend heavily on accessible waste heat for regeneration, and insufficient heat supply will greatly reduce their overall operational benefits.

McCutcheon and McGinnis [44,45] prepared draw solutions by mixing ammonium hydroxide (NH_4_OH) and ammonium bicarbonate (NH_4_HCO_3_) at specified ratios, and verified that such draw solutions can be efficiently recovered via distillation using low-grade heat sources. This is attributed to the thermal decomposition of NH_4_HCO_3_ into NH_3_ and CO_2_ at elevated temperatures. Among various draw solutes, ammonium bicarbonate has the lowest water solubility, coupled with a small molecular weight, leading to the highest reverse salt diffusion flux [46]. Additionally, relevant studies have demonstrated that NH_4_HCO_3_ tends to decompose rapidly when the operating temperature exceeds 30 °C [47,48]. It should be emphasized that the energy-saving advantage of this type of gaseous draw solutes largely relies on the on-site availability of waste heat or low-grade heat sources for thermal regeneration. Without accessible low-quality heat, the overall energy consumption will increase significantly and weaken its technical superiority.

### 3.2. Organic Compound Draw Solutions

Organic compounds have been extensively investigated and applied as draw solutes for forward osmosis. Although most organic draw solutes are non-ionic, their high water solubility enables the generation of considerable osmotic pressure [48]. Generally, organic draw solutes possess relatively large molecular weights, which can substantially suppress reverse solute diffusion. Even if minor reverse solute permeation occurs, it will not deteriorate the salinity of the feed solution.

Compared with inorganic salts such as sodium chloride (NaCl) and magnesium chloride (MgCl_2_), organic salts exhibit higher rejection rates but lower permeate flux during reverse osmosis concentration under identical operating conditions. Furthermore, organic draw solutions can be easily reconcentrated via nanofiltration or ultrafiltration systems. Notably, some organic draw solutes like glucose can be directly utilized without additional reconcentration and recovery, thus attracting extensive research attention.

Typical organic draw solutions include glucose, sucrose, ethanol, sodium formate, sodium acetate, sodium propionate, and switchable polarity solvents (SPS), among others. Among various organic draw solutes, there is an obvious distinction in application modes. Some organic substances, such as glucose and sucrose, can be used directly without additional separation or reconcentration processes, which greatly simplifies the system operation. By contrast, most synthetic organic solutes and switchable polarity solvents require extra membrane separation or distillation treatment to achieve regeneration before reuse.

In terms of applications, glucose and sucrose are widely used for drinking water purification and domestic wastewater treatment due to their non-toxic property and easy operation [49,50]. Organic salts such as sodium formate and sodium acetate are mostly applied in industrial low-salinity wastewater treatment and brine pre-concentration. Switchable polarity solvents are mainly adopted for complex organic wastewater disposal and resource recovery processes, showing unique advantages in difficult-to-treat water bodies.

Idaho National Laboratory has conducted systematic tests on novel draw solutions based on SPS [51,52,53,54]. SPS refers to a mixture composed of carbon dioxide, water and tertiary amines, and its prominent characteristic is the reversible polarity conversion between polar and non-polar states triggered by the presence or absence of CO_2_. The polarity conversion follows the chemical reaction below: NR_3_ + CO_2_ + H_2_O ⇌ HNR_3_^+^ + HCO_3_^−^. In addition, Yang et al. [55] explored the forward osmosis performance of sodium polyacrylate as a draw solute. The results revealed that the water flux of sodium polyacrylate draw solution was slightly higher than that of NaCl draw solution with the same osmotic pressure, while its reverse solute flux was merely one-tenth of the latter, indicating a remarkably low reverse solute diffusion.

### 3.3. Inorganic Compound Draw Solutions

Most forward osmosis investigations employ soluble inorganic compounds as draw solutes, which form homogeneous draw solutions and have been widely applied in laboratory and pilot-scale FO systems. Inorganic draw solutes mainly refer to electrolyte solutions, with a small quantity of non-electrolyte solutions also utilized. Inorganic compound draw solutions are favored predominantly owing to their high water flux, and they can be easily recovered via reverse osmosis or nanofiltration, or directly utilized without subsequent post-treatment [56,57,58].

Inorganic salts generally feature low molecular weights, abundant sources and low costs, making them ideal candidates for draw solutes as long as they exhibit high water solubility and can generate considerable osmotic pressure. Typical inorganic salts employed as draw solutes include NaCl, MgCl_2_, KNO_3_, MgSO_4_, KCl, Na_2_SO_4_, NaNO_3_, Ca(NO_3_)_2_, (NH_4_)_2_SO_4_, and other soluble electrolytes. Although small-molecule inorganic salt draw solutions boast the merits of high osmotic pressure and large water flux, they suffer from the critical drawback of severe reverse solute diffusion. Therefore, addressing the reverse permeation of inorganic salts during the FO process is the core issue for the screening and optimization of inorganic draw solutions.

Sodium chloride (NaCl) is the most widely used inorganic draw solute. Its predominant advantages lie in the sufficient natural reserves: seawater serves as a natural and low-cost draw solution resource. Additionally, NaCl exhibits high water solubility and excellent osmotic performance and can be readily concentrated via reverse osmosis. With the continuous expansion of research, researchers have attempted to employ low-toxicity macromolecular compounds as draw solutes in relevant studies. For instance, Boo and Elimelech et al. [59] conducted FO experiments using a carbon dioxide-trimethylamine (TMA) system as the draw solution. Compared with the ammonia-carbon dioxide draw solution, the larger molecular size of TMA effectively reduces reverse solute flux. Moreover, this draw system features a low thermal separation temperature, resulting in low energy consumption for reconcentration. Nevertheless, the small diffusion coefficient of TMA induces severe concentration polarization, which leads to a decline in water flux. Furthermore, TMA is toxic to the environment and human health, so its application is restricted to industrial scenarios only.

### 3.4. Magnetic Nanoparticle-Based Draw Agents

The application of magnetic nanoparticles (MNPs) as draw solutes in forward osmosis has attracted increasing research attention [60,61,62,63]. Warne et al. [61] first proposed the utilization of MNPs as novel draw solutes for the FO process. MNPs consist of a magnetic core and hydrophilic organic ligands coated on the core surface. The magnetic core endows the nanoparticles with magnetic responsiveness, enabling facile separation from aqueous solutions under an external magnetic field, while the grafted organic ligands contain abundant hydrophilic groups, rendering the MNPs highly soluble in water. Accordingly, MNP-based draw solutions can generate high osmotic pressure and deliver satisfactory water flux.

Ling et al. [62] synthesized thermo-responsive MNPs, which realized simple and energy-efficient regeneration. When the diluted MNP solution is heated above its critical temperature, the MNPs undergo spontaneous agglomeration and can be subsequently separated from the product water via ultrafiltration or a low-intensity magnetic field. Ge and Chung et al. [64,65] modified superparamagnetic nanoparticles with polyethylene glycol diacids of different molecular weights via thermal decomposition, and employed the modified MNPs as draw solutes for FO experiments; however, particle agglomeration was observed during the tests. In subsequent research, they adopted alkaline sodium polyacrylate polymer solution as the draw solute, which effectively overcame the agglomeration issue of magnetic particles, paving a new pathway for the development of high-performance draw solutions.

Compared with conventional draw solutions, magnetic nanoparticle-based draw solutions possess numerous outstanding advantages: a high specific surface area allows abundant hydrophilic organic ligands to be modified on the particle surface; the favorable magnetic responsiveness enables efficient separation via an external magnetic field; and the large particle size effectively suppresses reverse draw solute leakage. However, this type of draw solution also has inherent drawbacks, such as a tendency to aggregate and severe internal concentration polarization, both of which lead to the decline of water flux. Apart from particle aggregation and severe internal concentration polarization, this type of draw agent also faces other practical risks. Long-term operation may cause poor colloidal stability and a gradual loss of nanoparticles. In addition, the uniform dispersion and stable operation of magnetic nanoparticle suspensions are difficult to maintain during large-scale amplification, which becomes another key obstacle for industrial promotion. Therefore, developing stable, well-dispersed magnetic nanoparticle-based draw solutions with high osmotic pressure remains one of the critical challenges for researchers.

### 3.5. Polymer Gel Draw Agents

Polymer gels are three-dimensional crosslinked network materials formed via physical interactions or chemical covalent bonds, with particle sizes ranging from 50 to 150 μm. These materials can absorb and release water under external environmental stimuli, including temperature, pressure and light irradiation. Distinct from other draw solutes that need to be dissolved or dispersed in water to form draw solutions for FO applications, polymer hydrogels can act as draw agents directly by contacting the FO membrane without requiring water as a medium.

**Figure 2:**
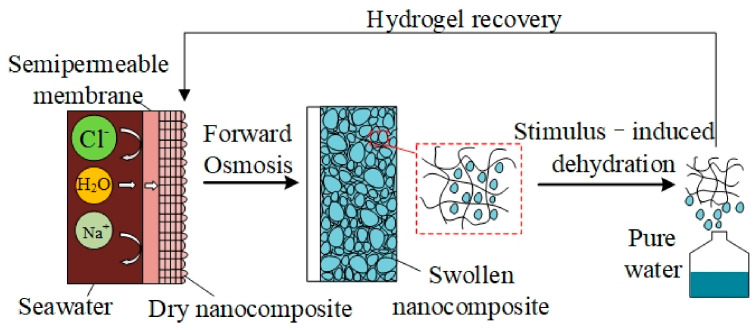
Schematic Diagram of FO Desalination Process Driven by Polymer Hydrogels. The process relies on osmotic water uptake and hydrogel swelling; pure water is subsequently recovered via thermal, mechanical or light stimuli according to hydrogel properties. (Adapted from Ref. [67]).

The polymer gel particles tightly adhere to the surface of the forward osmosis membrane and swell upon water absorption. After reaching swelling saturation, pure water can be recovered from the swollen hydrogels via heating or mechanical extrusion. The schematic diagram of the FO desalination process driven by polymer hydrogels is shown in Figure 2. Li et al. [66] found that a composite mixture of polymer gels and light-absorbing carbon materials could improve heat absorption and dehydration efficiency. Additionally, carbon filler particles can be incorporated into polymer gels to enhance the swelling ratio of hydrogels when used as FO draw agents [67]. Zeng et al. [68] introduced a small amount of reduced graphene oxide into hydrogels, which effectively improved the swelling performance of hydrogels and significantly enhanced the water flux of forward osmosis membranes. Pure water is mainly recovered from water-swollen polymer gels through heating, extrusion and other mild methods. Since polymer gels are insoluble in water and non-volatile, the recovered water features high purity, enabling efficient desalination via the FO process.

Besides the aforementioned draw solutions, various novel draw systems have also been developed, including micellar solutions, dendrimers, sodium polyacrylate polyelectrolytes (PAA-Na), acyl-TAEA, reverse osmosis brine, polymer-graphene composites, hexavalent phosphazene salts, and other emerging draw solutes.

### 3.6. Comparative Summary of Draw Solute Performance

In spite of diverse alternatives for draw solutes and draw agents, the regeneration of diluted draw media is a universal challenge. Most regeneration processes require additional energy input, which offsets the energy-saving superiority of FO to a certain extent. In addition, reverse solute leakage from draw solutions to feed solutions caused by reverse solute flux also deteriorates the overall separation performance.

As summarized in Table 3, each type of draw medium has distinct advantages and inherent defects. Inorganic salts are the most widely used draw solutes because of their high osmotic pressure and low cost, but their prominent reverse flux will contaminate the feed solution. Gaseous solutes feature low regeneration energy consumption, while organic solutes effectively inhibit reverse leakage. For emerging solid draw agents, including magnetic nanoparticles and polymer gels, they achieve nearly zero reverse flux, which is a major breakthrough. Nevertheless, their application is still restricted by material stability and mass transfer limitations.

## 4. Forward Osmosis Membrane Fouling

Membrane fouling refers to the accumulation of foulants on the membrane surface or within membrane pores, which is one of the major challenges in membrane separation processes. It not only causes a significant decline in water flux but also degrades the quality of the produced water. Severe membrane fouling requires intensive chemical cleaning or membrane module replacement to maintain stable membrane operation, resulting in increased treatment costs.

Compared with traditional pressure-driven membrane processes, forward osmosis membrane fouling shares certain similarities, such as the influence of solution chemistry, hydrodynamic conditions, and membrane surface properties, while also exhibiting unique fouling behaviors and mechanisms. Due to the absence of external hydraulic pressure, FO technology suffers from milder membrane fouling compared to pressure-driven membrane separation technologies. However, the potential impacts of membrane fouling caused by long-term operation cannot be ignored. Therefore, it is crucial to investigate the behaviors, influencing factors, and control strategies of forward osmosis membrane fouling.

Based on the type of foulants, forward osmosis membrane fouling can be mainly classified into four categories: inorganic fouling, organic fouling, colloidal fouling, and biofouling (Table 4) [69,70,71,72,73].

### 4.1. Inorganic Fouling

Inorganic fouling is primarily caused by the deposition of insoluble scaling on the FO membrane surface, which usually occurs when the feed solution is concentrated. The formation of insoluble scaling is mainly attributed to the salt concentration exceeding its saturation limit. Consequently, scaling substances represented by CaSO_4_, CaCO_3_, BaSO_4_, Ca_3_(PO_4_)_2_, and other insoluble salts deposit on or near the FO membrane through heterogeneous crystallization or deposition crystallization, leading to membrane scaling [74].

Common antifouling approaches include feed water softening, pH adjustment, and scale inhibitor addition, which effectively reduce supersaturation and suppress crystal nucleation on the membrane surface [75]. Surface hydrophilic modification and nanoparticle doping can also improve membrane anti-scaling performance by reducing ion adsorption and heterogeneous nucleation sites. For already formed inorganic scales, physical cleaning such as enhanced cross-flow flushing and air scouring can remove loose and newly formed inorganic deposits with low operational cost and no chemical damage. For dense and crystalline inorganic fouling layers, chemical cleaning using acid solutions, including hydrochloric acid, citric acid, and sulfuric acid, can efficiently dissolve mineral precipitates and recover membrane flux. In general, physical cleaning is environmentally friendly and protects membrane integrity but is only effective for reversible and loose inorganic fouling, while acid chemical cleaning achieves high removal efficiency for stubborn scaling but requires strict concentration control to avoid membrane degradation and material corrosion.

### 4.2. Organic Fouling

Organic fouling refers to the deposition of organic pollutants, typically represented by sodium alginate and humic acid, on the surface of forward osmosis membranes. Mi et al. were the first to investigate the FO membrane fouling behaviors and cleaning efficiency of three model pollutants, namely sodium alginate and humic acid. Their findings indicated that organic fouling is associated with the interaction of intermolecular adhesion forces, and hydrodynamic conditions (osmotic resistance, shear force) are the main factors governing the development of organic fouling [76].

Additionally, the presence of a large amount of divalent ions (e.g., Ca^2+^, Mg^2+^) in the solution often leads to more severe membrane fouling, which is mainly due to the strong interactions between divalent ions and organic pollutants.

It should be noted that it is inappropriate to regard mild single organic fouling as an inherent absolute advantage in practical applications. In actual long-term operating systems, mixed fouling and synergistic effects among different foulants are prevalent, which will cause severe water flux decline and operational deterioration. Divalent cations such as Ca^2+^ can act as bridging agents to tightly combine alginate, humic substances and colloidal particles, forming dense composite fouling layers on the membrane surface. Meanwhile, extracellular polymeric substances (EPS) secreted by microbial communities further strengthen the adhesion of foulants and accelerate the formation of stable biofilms. The synergistic interaction of inorganic, organic, colloidal and biological pollutants greatly aggravates membrane fouling, and this problem becomes more prominent with the extension of operation time.

Common preventive strategies of organic fouling include feed pretreatment by coagulation, adsorption, and ultrafiltration, which significantly reduce organic loading before the FO stage [70,76]. Membrane surface modification, including hydrophilic coating and charge regulation, can weaken organic adsorption and improve long-term antifouling stability. For reversible organic fouling, cross-flow washing and hydraulic backwashing can effectively remove loosely adsorbed organic matter and restore most water flux [77]. Irreversible organic fouling requires chemical cleaning using alkaline solutions, surfactants, or oxidizing agents such as sodium hypochlorite. Alkaline cleaning hydrolyzes organic macromolecules and destroys hydrophobic adsorption layers, while oxidants decompose complex organic structures into small soluble fragments. These chemical methods exhibit high removal efficiency for aged organic fouling; however, excessive oxidation or long-term alkaline exposure may gradually damage the polyamide active layer and reduce membrane service life.

### 4.3. Colloidal Fouling

Colloidal fouling is mainly induced by colloidal substances (e.g., silica nanoparticles) in the solution, which affect the osmotic flux through two side effects: increasing hydraulic resistance and hindering the diffusion of solutes in the cake layer. Studies by Park et al. [78] have shown that the concentration polarization-enhanced osmotic pressure caused by the cake layer formed during colloidal fouling is the primary reason for the decline in FO water flux. It should be noted that organic fouling generally refers to membrane fouling caused by alginates, humic acids, proteins, and other organic substances during the FO process. Notably, there is usually a synergistic effect between the aforementioned different types of fouling, and their combination will exacerbate the membrane fouling situation.

For colloidal fouling, the most effective preventive methods are pre-filtration, coagulation-sedimentation, and inline microfiltration pretreatment, which largely eliminate colloidal particles before membrane filtration [78]. Additionally, membrane surface charge modification can weaken electrostatic adsorption and inhibit colloidal deposition. For removable colloidal cake layers, high-shear cross-flow flushing and periodic physical washing can achieve excellent cleaning effects with nearly zero membrane damage. For compacted colloidal fouling formed by long-term operation, combined acid and neutral surfactant cleaning can disperse aggregated colloids and recover membrane permeability. Physical cleaning strategies for colloidal fouling are highly repeatable and suitable for frequent maintenance, while chemical cleaning is only required for irreversible compacted layers, balancing cleaning effectiveness and membrane protection.

### 4.4. Biofouling

**Figure 3:**
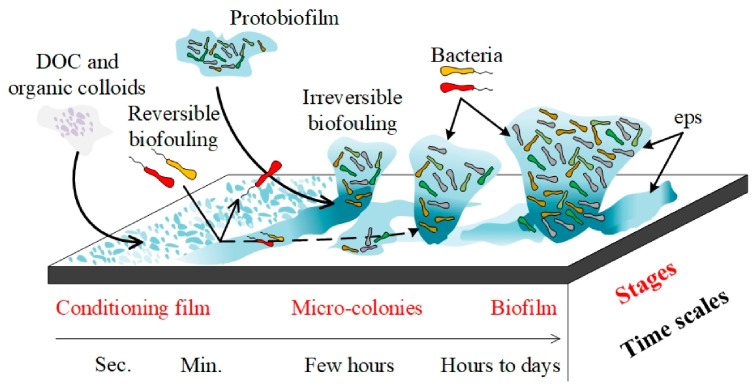
Schematic illustration of biofouling development and biofilm maturation. The diagram presents an ideal sequential process of conditioning film, reversible adhesion, microcolonies, irreversible adhesion and EPS-rich mature biofilm. Note that partial stages may overlap under real operational conditions. (Adapted from Ref. [79]).

Biofouling refers not only to the deposition of microorganisms on the membrane surface but also to the formation of biofilms resulting from the reproduction of microorganisms on the membrane surface. Biofouling is ubiquitous in membrane filtration processes and is also the most difficult to control. The formation of biofilms on the membrane surface is a phased process, but it can also form simultaneously, as illustrated in Figure 3 [79].

Firstly, a primary fouling layer composed of precursor organic substances such as proteins, lipids, and polysaccharides, as well as humic substances, nucleic acids, and amino acids, is formed on the membrane surface. The formation of this primary fouling layer facilitates the adhesion of microorganisms. Secondly, bacteria in the feed solution reversibly adhere to the membrane surface through the action of van der Waals forces, hydrogen bonds, and other intermolecular interactions. At the same time, macromolecular organic substances deposit on the membrane surface, promoting the reproduction of bacteria and the formation of biofilms on the membrane surface. Subsequently, bacteria and pollutants continuously deposit on the membrane surface, thereby forming a biofilm composed of bacteria, proteins, polysaccharides, and other substances on the membrane surface [80].

For biofouling, common antifouling strategies include membrane antibacterial modification, UV pretreatment, and periodic disinfection of feed water, which effectively inhibit initial bacterial colonization and biofilm development [68]. Functional coatings with antibacterial agents can significantly reduce microbial adhesion and improve long-term biofouling resistance. For immature and reversible biofouling, cross-flow hydrodynamic cleaning can remove loosely attached microbial colonies. However, mature biofilms with dense EPS matrices are highly resistant to physical washing and require chemical cleaning with biocides, chlorine solutions, or enzymatic cleaners. Chlorination can effectively inactivate microorganisms and degrade EPS structures, while enzymatic cleaning specifically decomposes extracellular polymeric substances with minimal membrane damage. Although chemical disinfection achieves high biofouling removal efficiency, frequent chlorine exposure may degrade membrane active layers, and residual biocides may cause secondary water pollution. Therefore, combined physical–chemical maintenance strategies are currently considered the most stable and effective approach for long-term FO system operation.

## 5. Applications and Techno-Economic Assessment of Forward Osmosis Technology

With the development of FO membrane materials and draw solutions, forward osmosis membrane technology has been widely applied in numerous fields, including seawater desalination, wastewater treatment and reuse, pressure-retarded osmosis for power generation, and energy development, owing to its unique advantages such as low membrane fouling propensity, low energy consumption, high rejection, and high water recovery.

### 5.1. Seawater Desalination

As early as the 1960s, forward osmosis was proposed for seawater desalination. However, due to insufficient technological maturity at the time, FO did not attract widespread attention, and reverse osmosis was more extensively applied instead. In the RO membrane process, hydraulic pressure is applied to overcome osmotic pressure, forcing fresh water through the membrane material. In contrast, in the FO process, the osmotic pressure gradient drives fresh water from the feed solution side to the draw solution side. The osmotic driving force in FO is significantly greater than the hydraulic driving force in RO; therefore, FO can substantially reduce costs in desalination applications.

HTI developed an FO-PRO (forward osmosis–pressure retarded osmosis) process that utilizes the osmotic pressure generated by an FO module to drive RO seawater desalination, thereby eliminating the need for external energy input while producing water of higher quality than that from conventional RO. McCutcheon et al. [44] used a cellulose acetate (CA) membrane and an ammonia/carbon dioxide draw solution. When seawater was used as the feed solution, the osmotic pressure reached as high as 200 bar, the water flux reached 25 L/(m^2^·h), and the salt rejection exceeded 95%. Compared with conventional seawater desalination technologies, energy savings of approximately 72–85% were achieved. McGinnis and Elimelech [45] employed ammonium bicarbonate as the draw solution and used a distillation column for draw solution reconcentration. They found that the energy consumption for seawater desalination using FO was reduced by 72.1% compared with RO desalination. Tan and Ng [57] developed a coupled FO–NF/NF system and demonstrated that it could save 25% of energy compared with two-stage RO seawater desalination. In September 2012, Modern Water plc commissioned the world’s first commercial forward osmosis desalination plant at Al Najdah in the Al Wusta region of Oman [81]. This commercial-scale plant has a production capacity of 200 cubic meters per day and was built under a contract with Oman’s Public Authority for Electricity and Water. Compared with conventional RO, this plant achieved lower operating costs, reduced energy consumption (approximately 30–42% energy savings depending on feed water quality), and improved membrane fouling resistance and membrane lifespan.

It is important to note, however, that FO-based seawater desalination remains less mature than RO at the industrial scale, and the Oman plant represents a niche commercial application rather than widespread industrial adoption. The most prominent bottleneck is its inherently low water flux compared with pressure-driven membrane processes, which requires a huge membrane area to guarantee equivalent water production capacity and pushes up initial investment. In addition, the regeneration and reconcentration of diluted draw solutions consume substantial additional energy and operating costs, largely offsetting the energy advantages of the FO main process. Moreover, persistent reverse solute flux causes continuous loss of draw solutes and deteriorates feed water quality, further increasing operational expenditure. These technical and economic drawbacks collectively hinder the widespread promotion of FO in commercial seawater desalination projects, even though it shows excellent performance under laboratory and small pilot conditions.

### 5.2. Pressure-Retarded Osmosis (PRO) Power Generation

Pressure-Retarded Osmosis (PRO) is a form of forward osmosis in which an external pressure less than the osmotic pressure is applied to the concentrated solution side, allowing water molecules to still permeate from the dilute solution side to the concentrated solution side. The osmotic pressure difference between seawater and freshwater is approximately 2.7 MPa, which represents the theoretical upper limit of available energy. However, only a fraction of this potential energy can be harvested in practical PRO systems, resulting in relatively low actual power densities. Though PRO can achieve osmotic energy conversion, the technology still faces prominent technical bottlenecks, including insufficient mechanical strength of membranes under long-term pressurization, severe membrane fouling caused by complex water components, and low overall energy conversion efficiency. Compared with traditional fossil energy, osmotic energy is a renewable and environmentally friendly energy source. Meanwhile, compared with other new marine energy sources such as tidal energy, temperature difference energy, and biomass energy, osmotic energy has a higher energy density.

**Figure 4:**
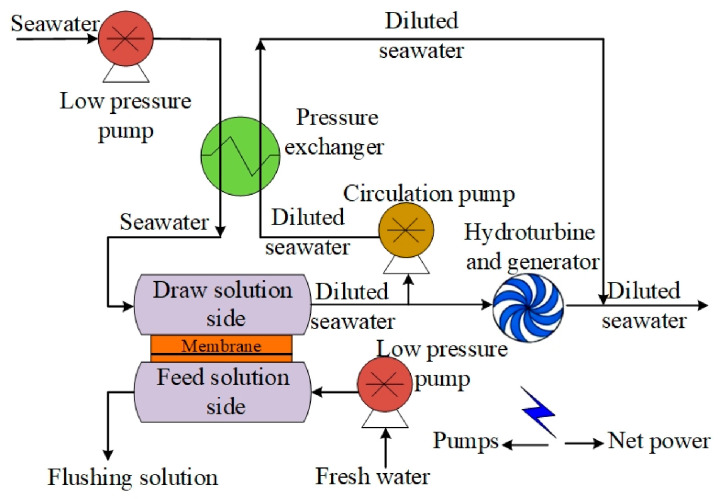
Pressure-Retarded Osmosis (PRO) system. Adapted from Ref. [82].

At present, PRO technology has advanced from laboratory research to pilot-scale testing and early demonstration projects (Figure 4) [82]. As a leader in osmotic energy development, Statkraft built the world’s first osmotic power prototype pilot plant in Tofte, Norway, which was officially opened in November 2009. This pilot-scale prototype, developed together with Sintef, was designed primarily for technology validation and continuous 24-h operation testing [83]. In this process, seawater was used as the draw solution, the osmotic pressure difference across the membrane was 12.5 bar, and the achievable power density was 5 W/m^2^. Critically, these experimentally realized power densities remain drastically lower than the theoretical upper limits calculated from ideal osmotic pressure gradients, leaving substantial room for membrane and system optimization before economically viable power output can be attained. It should be emphasized that this plant was a prototype (not a commercial facility), and Statkraft subsequently shelved its osmotic power investments due to economic and technical challenges [84].

In 2013, Japan’s Megaton Water Supply System project used the concentrated brine produced by RO as the draw solution for power generation. This project adopted hollow fiber PRO membrane modules and achieved a power density of 13.3 W/m^2^ under an osmotic pressure difference of 30 bar [85]. This demonstration project developed a CTA hollow-fiber PRO membrane module and conducted continuous operation testing at a prototype plant for over one year. The Netherlands’ first salinity gradient energy pilot power plant was put into operation in 2015, capable of treating 220,000 L of seawater and fresh water per hour, respectively [86]. In addition, osmotic energy is not limited to power generation from the mixing of fresh water and seawater. Bobert et al. used ammonia-carbon dioxide as the draw solution and converted low-value waste heat energy into electrical energy through the forward osmosis process, which is called an osmotic heat engine [87].

The reported power density of 5–13 W/m^2^ in existing PRO pilot tests is far from the technical and economic threshold for commercial power generation. In practical engineering, a power density of 200–300 W/m^2^ is generally regarded as the minimum standard to realize profitable operation. Apart from insufficient power output, PRO systems are also troubled by internal concentration polarization, severe membrane fouling under long-term running, and high costs for draw solution circulation and membrane maintenance. These critical gaps between current technical indicators and commercial requirements mean that PRO is still limited to laboratory research and demonstration pilots, and major technological breakthroughs are still needed before it can be applied for industrial power production.

### 5.3. Wastewater Treatment and Reuse

Wastewater contains complex pollutants, which are prone to causing membrane fouling during the membrane treatment process. Due to its driving force based on osmotic pressure difference and low membrane fouling propensity, forward osmosis technology has been widely applied in various water treatment fields, including municipal wastewater treatment, landfill leachate treatment, and forward osmosis membrane bioreactors. Most relevant studies on FO-based municipal wastewater treatment are currently conducted at laboratory and bench scales. A small number of on-site pilot tests have been carried out, but full-scale industrial applications are still rare.

#### 5.3.1. Municipal Wastewater Treatment

Forward osmosis has significant potential application value in wastewater treatment and resource recovery. Featuring a high rejection rate, FO can recover nutrients in concentrated wastewater in the form of fertilizer production, among which the Direct Forward Osmosis (Direct FO) process is one of the commonly used technologies in wastewater treatment and reuse.

Holloway et al. used forward osmosis to concentrate anaerobic digestion effluent, and the results showed that nitrogen and phosphorus nutrients achieved high rejection rates, while the water flux recovery rate reached 70% [88]. Zhang et al. [89] proposed a Direct FO process for municipal wastewater treatment using seawater as the draw solution. The diluted seawater can be reused via RO or directly discharged, while the concentrated municipal wastewater can be subjected to anaerobic digestion for energy recovery. The results indicated that the COD of municipal wastewater was concentrated by more than 300%. Ansari et al. [90] used a CTA reactor to treat the effluent from the primary sedimentation tank of a wastewater treatment plant. Under 6 MPa, the volume of wastewater could be reduced to 10% of that before treatment, and the COD of the wastewater reached more than 2000 mg/L, facilitating subsequent anaerobic digestion.

In addition, forward osmosis has the unique property of reverse diffusion. Researchers used MgCl_2_ as the draw solution, which not only provided the driving force for concentrating nutrients in wastewater but also utilized the reverse diffusion of MgCl_2_ to provide Mg sources for the formation of struvite fertilizer [91,92]. The results showed that the wastewater was concentrated 5 times, and the concentrations of nutrients ammonia nitrogen and phosphorus reached 1210 mg/L and 615 mg/L, respectively, laying a foundation for the formation of struvite.

#### 5.3.2. Landfill Leachate Treatment

Landfill leachate is generated by landfilled solid waste, with complex and variable components. Traditional wastewater treatment methods cannot effectively remove total dissolved solids (TDS) from landfill leachate, and pollutants such as soluble heavy metals and organic substances contained in landfill leachate will affect the biological treatment process [93].

Coday et al. [94] investigated the feasibility of FO for landfill leachate treatment, and the results showed that after FO treatment, the TDS of the effluent from landfill leachate was lower than 100 mg/L. Sun et al. [95] studied the efficiency of the combined process of “Fenton oxidation + membrane absorption + FO” for leachate treatment. The Fenton method removed a large amount of organic substances and suspended solids from landfill leachate, and the effluent was treated by membrane absorption to remove most of the NH_3_-N (removal rate of 91.8%), and the (NH_4_)_2_SO_4_ generated in the acid absorption solution was recovered and reused as fertilizer. Subsequently, the FO system was further adopted for advanced treatment to achieve the discharge standard of the effluent, while the concentrated water generated by the FO system could be backfilled into the landfill after low-temperature evaporation, solidification and drying. Another study from South Korea used FO to concentrate landfill leachate (Suwon Landfill in South Korea) [96]. The system adopted an ultraviolet (UV)/H_2_O_2_ advanced oxidation process, which activated H_2_O_2_ through UV to generate more ·OH to oxidize organic substances in the leachate. The leachate oxidized by UV/H_2_O_2_ was then concentrated by FO.

In general, industrial practices of forward osmosis for landfill leachate treatment are very few compared with other technologies. From the existing pilot-scale data, forward osmosis has broad application prospects in landfill leachate treatment.

#### 5.3.3. Osmotic Membrane Bioreactor (OMBR)

The osmotic membrane bioreactor (OMBR) is another commonly used FO treatment process. It employs FO membranes to replace the microfiltration (MF) or ultrafiltration (UF) membranes in traditional membrane bioreactors (MBR), and is a wastewater treatment system integrating the retention effect of forward osmosis membranes and the degradation effect of activated sludge. Compared with traditional MBR, OMBR replaces the UF or MF membranes in MBR with FO membranes.

OMBR has the following technical and operational advantages: (1) FO operates under low pressure or no external pressure, thus exhibiting low potential membrane fouling and low system energy consumption; (2) the effluent quality of the system is excellent; (3) the diluted draw solution is reused after reverse osmosis treatment, so no concentrated water is generated and the pure water recovery rate is higher [97,98,99]. Alturki et al. [100] investigated the removal of 50 trace organic chemicals (TOrCs) by OMBR, and the results showed that the removal rate of TOrCs with a molecular weight greater than 266 g/mol was all above 80%. Due to the small pore size of FO membranes, they have a high rejection rate for TOrCs with large molecular weights and increase the residence time of TOrCs in OMBR, thereby improving the removal rate.

OMBR is a wastewater treatment process with good prospects. However, systematic studies on the specific wastewater treatment effect and membrane fouling of OMBR still need further improvement.

### 5.4. Application of Forward Osmosis in the Energy Field

#### 5.4.1. Shale Gas Development

Shale gas is an important unconventional natural gas resource, and its exploration and development are in a stage of rapid development. The wastewater generated during shale gas development contains a large amount of soluble substances. The total dissolved solids (TDS) of produced water in the initial stage of exploitation varies greatly, with some less than 5000 mg/L, while the TDS of high-salinity water may exceed 100,000 mg/L.

In recent years, with the development of forward osmosis membranes, researchers have carried out studies on the treatment of wastewater from shale gas development using FO membrane processes. Among them, HTI and Oasys Water have vigorously promoted the application of FO in the field of shale gas wastewater. HTI’s “Green Machine” series technologies for shale gas wastewater have completed multiple field pilot tests. HTI has developed the first and second generations of the “Green Machine” shale gas wastewater treatment technology, with a water recovery rate of up to 85%. Moreover, after the second-generation “Green Machine” concentrated shale gas wastewater five times, the water flux only decreased by 18% without any membrane cleaning [94]. Hickenbottom et al. [101] investigated the treatment of shale gas wastewater by the FO process under different operating conditions. The results showed that the FO membrane had a high rejection rate for organic substances and inorganic ions in the wastewater, the water recovery rate could reach more than 80%, and the recovery rate of water flux by osmotic backwashing was discussed. In addition, in the Marcellus shale gas extraction area, pilot-scale studies have confirmed that the forward osmosis-distillation process can obtain pure water, and the TDS of the wastewater decreased from 73,000 ± 4200 mg/L to 300 ± 115 mg/L [102].

Overall, forward osmosis technology has obvious potential advantages in shale gas wastewater treatment and can significantly reduce energy consumption.

#### 5.4.2. Mining Development

A large amount of wastewater is generated during mining processes, especially coal mining. Such wastewater is usually strongly acidic or alkaline, with high salinity and trace metal elements, and must be treated before discharge. For mining wastewater, there are generally the following types of treatment methods: limestone neutralization, chemical precipitation, and passive treatment processes [103]. In mining operations in Australia, some mines have begun to use RO technology for wastewater treatment. However, there are very few cases of using FO in mining wastewater treatment. The FO-RO coupling process for mining wastewater is still in the laboratory research stage in most regions.

According to the research on FO application in mining wastewater treatment, the FO-RO coupling process is mainly used to treat mining wastewater. This method can avoid the high-cost pretreatment process required by traditional RO and reduce the cost of the entire desalination process. The Commonwealth Scientific and Industrial Research Organisation (CSIRO) of Australia carried out a laboratory-scale study on the FO-RO process with a flux of 3–5 L·m^−2^·h^−1^, successfully treating mining wastewater and obtaining reusable substances.

Overall, the application of the FO-RO coupling process for mining wastewater treatment is expected to become the best choice for low-cost treatment in this industry.

#### 5.4.3. Lithium Extraction from Geothermal Water

Hydrothermal geothermal energy is a resource combining “heat, water, and minerals”. It is not only a clean, safe, and renewable energy source but also a mineral resource rich in elements such as lithium, rubidium, cesium, and boron. To begin with, abundant lithium-containing geothermal brines have been discovered around the world, providing rich raw materials for lithium resource development. Lithium is an important strategic metal, and research on the development and utilization of lithium resources has been increasing worldwide. Geothermal water, especially high-temperature geothermal water, generally contains a certain amount of lithium. Current research on FO-based geothermal lithium extraction is mainly concentrated in laboratories and on small pilot scales. For example, the lithium content in North American hot springs exceeds 25 ppm, that in Arima Hot Spring in Hyogo Prefecture, Japan, reaches 55.8 ppm, that in geothermal water in Cornwall Mines, UK is 260 mg/L, and the average lithium content in hot springs and geothermal wells in Tibet, China is 11.4 mg/L. At present, brine lithium extraction is mainly dominated by lithium extraction (lithium extraction from salt lakes), whose development has become mature worldwide and has formed an important industry. However, only a small number of experimental studies on lithium extraction from geothermal brine have been carried out [104,105,106].

Currently, there are more than 60 variants of direct lithium extraction technologies, but the basic process includes such as membrane-based ion concentration enrichment [107,108,109,110,111,112,113,114], which selectively collects only lithium, rubidium, and cesium chloride (the main form of lithium in brine); the separated water is injected underground through boreholes; the enriched concentrate is purified and further concentrated by nanofiltration and chemical precipitation to precipitate metal ions, producing lithium hydroxide or lithium carbonate for battery manufacturing [115]. Currently, several technically mature lithium recovery methods have been widely applied in industrial practice, including adsorption, ion exchange, solvent extraction and selective membrane separation. These approaches are the mainstream choices for commercial lithium production from brines [116,117,118,119]. At present, certain breakthroughs have been made in geothermal lithium extraction technology, and the construction of relevant demonstration projects is underway. The Karlsruhe Institute of Technology in Germany and the Ingler-Bunt Institute have jointly developed a geothermal lithium extraction process—the Grimmer–Saravia process. This process mainly consists of two steps: filtering lithium ions from hot water and further condensing until lithium precipitates in the form of salt, which is a combination of the membrane method and the condensation lithium precipitation process.

The thermal energy required in the FO process can directly utilize the low-grade thermal energy of geothermal tail water, realizing cascade utilization of energy and forming an energy synergy closed loop of “producing water with heat and extracting lithium with water”, which is highly economical and environmentally friendly. Forward osmosis technology has broad application scenarios in lithium extraction from geothermal water. At present, the research team has also designed and explored the technical path of lithium extraction from geothermal water using forward osmosis technology.

The treatment device for lithium extraction from geothermal water using forward osmosis technology consists of five parts: a pretreatment system, a forward osmosis treatment system, a nanofiltration draw solution circulation system, and a heat exchange system. Geothermal hot spring water is connected to the pretreatment device through a raw water pump (equipped with a pre-installed flat-sheet MBR) [120,121]. The pretreatment filtration precision is 0.2~0.4 μm to remove particulate impurities and suspended solids, and the temperature is reduced to 25 °C~30 °C to improve flux and protect the forward osmosis membrane module. The outlet of the pretreated wastewater is connected to the forward osmosis device, where pollutants are retained, water molecules permeate through the forward osmosis membrane module into the draw solution, and the diluted draw solution is recycled through the nanofiltration device, resulting in high enrichment of the raw water. The tail water after extraction is heated by a heat exchanger and injected underground by a high-pressure pump. In the tail water reinjection link, anti-corrosion coatings are used to protect the pipeline materials, which can effectively achieve corrosion resistance, oxidation resistance, wear resistance, and scaling resistance of the high-temperature geothermal system [122]. Meanwhile, during the reinjection process, chemical inhibitors can be used to prevent scaling of the geothermal well system according to the actual scaling situation.

In practical application configurations, FO lithium enrichment systems are mainly implemented through three mature technical routes verified in laboratory and pilot scales, including standalone single-stage FO concentration, multi-stage integrated membrane coupling processes, and hybrid biological-membrane systems for organic-containing brines. The standalone FO configuration generally adopts flat-sheet or hollow-fiber membrane modules with feed solutions of pretreated raw lithium brine and commonly used draw solutions such as magnesium chloride, sodium chloride and thermally decomposable ammonia–carbon dioxide mixed systems, where the active-layer-facing-feed (AL-FS) membrane orientation and counter-current flow mode are preferred to mitigate internal concentration polarization and improve the concentration factor, achieving a single-pass lithium concentration multiple of 2.5–4.0 for typical low-grade brines.

For high-purity lithium purification scenarios, the integrated nanofiltration–FO–membrane distillation coupling process is widely adopted, in which nanofiltration serves as the pretreatment unit to remove most magnesium ions and reduce the Mg/Li ratio, FO further concentrates the low-salinity permeate to greatly reduce the brine volume, and membrane distillation or waste heat-driven thermal regeneration realizes continuous recycling of diluted draw solution, while residual salinity gradient energy can be partially recovered through auxiliary PRO units to reduce overall energy consumption. For special organic-rich lithium-containing produced water, the FO-membrane bioreactor integrated system can simultaneously realize organic pollutant degradation and lithium brine concentration, effectively alleviating organic fouling and improving operational stability. In terms of membrane performance requirements specifically for lithium extraction applications, FO membranes need to comprehensively balance high water flux, low reverse solute flux, excellent ion selectivity and long-term anti-fouling stability under complex brine environments.

Huang et al. conducted a combined life cycle assessment (LCA) and techno-economic assessment (TEA) of lithium recovery from geothermal brine using lithium-aluminum-layered double hydroxide chloride (LDH) sorbent coupled with forward osmosis [123,124]. The analysis assumed that the lithium extraction unit is an add-on to a 50 MW geothermal power plant in California. LCA results indicate that, compared with conventional LiOH and Li_2_CO_3_ production pathways, this technology achieves a 1–95% reduction in environmental impacts. The add-on unit could achieve a payback period of less than 1 year, reaching net present values of $454M and $315M for LiOH and Li_2_CO_3_ production pathways, respectively. These favorable environmental and economic performances suggest that LDH sorption coupled with forward osmosis has great potential to enable the domestic production of battery lithium compounds. Additionally, Wang et al. developed a high-performance TFC-FO membrane specifically designed for lithium ion concentration in brine, addressing the critical need for FO membrane materials with high flux and high rejection rates for geothermal lithium concentration [125].

Current mainstream materials include thin-film composite polyamide membranes and modified cellulose triacetate membranes; qualified lithium-extraction FO membranes require a hydrophilic, low-tortuosity porous support layer to minimize internal concentration polarization, a dense and defect-free active layer to ensure high rejection of impurity ions such as magnesium and sodium, and surface hydrophilic modification to resist brine scaling and colloidal fouling.

In contrast, forward osmosis (FO) is an emerging technology for geothermal lithium extraction. It acts primarily as a concentration and process integration stage, rather than a standalone mature lithium extraction technology. Its practical application is still in the exploratory and pilot stage. Therefore, using forward osmosis technology to enrich and concentrate lithium ions in geothermal tail water, the high concentration multiple allows the stock solution to be directly used for chemical precipitation to prepare lithium carbonate, which can greatly optimize the lithium ion extraction process, reduce operating costs, and is expected to realize the rapid development of the geothermal lithium extraction industry.

### 5.5. Techno-Economic Analysis of FO Across Typical Applications

Techno-economic performance is a decisive factor determining whether forward osmosis can move from laboratory research to large-scale commercial deployment. This section analyzes capital investment, energy consumption, operational cost and economic competitiveness of FO in its major application fields, and makes horizontal comparisons with conventional competing technologies.

For seawater desalination, FO can reduce process energy consumption by 72–85% compared with traditional reverse osmosis (RO) in lab and pilot tests. However, its inherently low water flux requires a much larger membrane area to reach equivalent water output, which sharply raises the initial capital cost for membrane modules, frame and pipeline systems. In addition, the regeneration of diluted draw solution introduces an extra energy-consuming unit, and the continuous loss of draw solutes caused by reverse solute flux increases daily material cost. Overall, the total operational cost of FO seawater desalination is currently 15–30% higher than that of mature RO processes. FO only shows marginal economic advantages in small-scale scattered desalination projects equipped with abundant low-grade waste heat; for large centralized desalination plants, RO still maintains absolute cost superiority.

In pressure-retarded osmosis (PRO) for salinity gradient power generation, current pilot facilities deliver a power density of merely 5–13 W/m^2^, far below the 200–300 W/m^2^ threshold for profitable commercial operation. The high cost of high-performance FO membranes, frequent membrane replacement due to biofouling and inorganic scaling, plus the energy loss in draw solution circulation, make the levelized cost of electricity of PRO far higher than conventional power generation and renewable energy technologies. At present, PRO projects can only operate as technical demonstration platforms and cannot generate economic benefits independently.

When applied to municipal wastewater treatment and osmotic membrane bioreactors (OMBR), FO exhibits excellent effluent quality and strong anti-fouling ability under low operating pressure. Compared with traditional MBR, FO-based systems cut chemical cleaning frequency and extend membrane service life to a certain degree, lowering maintenance costs. Nevertheless, the additional draw solution preparation and regeneration system increases the footprint and overall energy consumption. From a full life-cycle perspective, FO-OMBR is economically viable for high-standard water reuse scenarios with strict discharge limits, but it is not cost-effective for conventional domestic wastewater treatment with ordinary discharge requirements.

For industrial wastewater treatment, including shale gas wastewater, mining wastewater and landfill leachate, FO is well-suited for high-salinity wastewater concentration due to its low-pressure operation. Compared with membrane distillation (MD) and electrodialysis (ED), FO avoids the risks of membrane wetting in MD and high electrode cost in ED. Its economic advantages become prominent when the project has on-site waste heat for draw solution regeneration. Without supporting heat resources, the high energy consumption of solute reconcentration will erase its technical merits.

In the emerging field of lithium extraction from geothermal brine, FO acts as an efficient pre-concentration unit to raise lithium concentration and reduce the treatment load of subsequent extraction processes. It effectively cuts the scale and investment of follow-up adsorption, ion exchange and solvent extraction units. At this stage, FO-coupled lithium extraction processes are still in a small pilot stage. Limited by immature matching technologies and a small market scale, the comprehensive cost is relatively high. But considering the high economic value of lithium resources, this combined process has great cost-reduction potential with further scale-up and technical optimization.

In summary, FO is not a universal cost-competitive technology at the current stage. It cannot fully replace mature separation technologies such as RO, MD and ED. Its economic advantages are concentrated in specific niche scenarios: projects equipped with sufficient low-grade waste heat, high-salinity wastewater treatment requiring mild operating conditions, and resource recovery projects with high added value. For general water treatment and large-scale desalination, FO still needs technological breakthroughs to reduce flux limitations and system costs before achieving widespread commercial promotion.

## 6. Challenges and Future Research Directions

### 6.1. Key Remaining Challenges

Overall, forward osmosis faces a series of inherent limitations that distinguish it from mature competing technologies, including reverse osmosis (RO), membrane distillation (MD) and electrodialysis (ED). First of all, low intrinsic water flux is a universal shortcoming of FO membranes, resulting in lower unit production efficiency than RO, the dominant technology for desalination and water treatment. Second, draw solution regeneration is an unavoidable energy-intensive unit operation in FO systems; the total energy consumption of the whole process is highly dependent on regeneration efficiency, while RO and MD directly produce product water without an extra solute recovery loop. Third, reverse solute flux is a unique challenge for FO, which leads to solute loss, feed water contamination and increased operating costs, a problem that does not exist in RO, MD and ED. In terms of fouling characteristics, FO suffers from specific internal concentration polarization coupled with surface fouling, and mixed fouling evolves more complexly in the porous support layer, making its fouling control and cleaning more difficult than pressure-driven membrane technologies.

From an economic perspective, RO has been fully industrialized with mature supply chains, low unit cost and stable long-term operation performance, occupying the mainstream market of desalination and wastewater reclamation. Membrane distillation adapts well to high-salinity brines and high-temperature water bodies, yet it also faces high energy consumption and membrane wetting risks. Electrodialysis performs well for ionic separation but is not suitable for organic pollutant removal. Compared with these alternatives, FO currently has higher comprehensive operational costs due to extra draw solution management and low flux. Although it possesses unique advantages such as low fouling tendency under mild operating pressure and flexible coupling with resource recovery processes, the large gap between laboratory performance and commercial economy remains the core obstacle restricting its large-scale application.

### 6.2. Future Directions

Forward osmosis (FO) technology is a promising treatment technology. With the technological progress of membrane materials and draw solutions, the application of FO technology will become increasingly widespread. However, FO technology is still not mature and perfect at present. For example, FO membranes still suffer from relatively low water flux compared with RO, limited varieties of commercial products, unsatisfactory pollutant rejection for specific contaminants, severe internal concentration polarization and persistent reverse solute flux. In terms of draw media, the regeneration of diluted draw solutes and draw agents often consumes extra energy, leading to increased operating costs. These technical and economic limitations, together with the relatively insufficient long-term operational data of full-scale projects, greatly restrict the large-scale commercialization of FO technology.

Therefore, in future research, the focus of FO technology research should be on the following aspects:

(1) Research and development of FO membranes. It is crucial to develop FO membranes with high mechanical strength, high water flux, and high salt rejection rate. In the development of asymmetric FO membranes, an important breakthrough is to develop new membrane support layer materials with high mechanical strength to reduce the thickness of FO membranes and improve the hydrophilicity of the support layer. In addition, the development of symmetric FO membranes with high mechanical strength is also of great significance.
(2) Research on new draw solution concentration methods. Draw solutions with high osmotic pressure can usually accelerate the water extraction rate, but at the same time, they will aggravate the diluted internal concentration polarization, which forms a contradiction between high water flux and large internal concentration polarization. Therefore, how to alleviate or even eliminate this contradiction is of great significance for the development of draw solutions. In addition to minimizing the required energy consumption, the draw solution reconcentration method should also explore the complementarity and synergy between the concentration process and other technologies.
(3) Expanding the application in the energy field, especially in the field of lithium extraction from geothermal water. The energy field plays a crucial role in the global economy and profoundly affects sustainable development. The FO process has better tolerance to colloids, organic substances and other pollutants in geothermal brine, which can effectively alleviate membrane fouling and ensure the long-term stable operation of the system. Therefore, future research should focus on developing efficient draw solution systems suitable for lithium extraction from geothermal water, optimizing the full-process integration of FO and lithium extraction processes, and promoting the iteration of FO technology from a “water treatment technology” to a “core technology for energy and resource recovery”.
(4) Exploring wider application fields. The optimal combination conditions of FO technology with other technologies still need to be explored. Efforts should be made to expand its application in fields such as osmotic drug delivery systems, wastewater treatment under large-scale operation conditions, and pressure-assisted osmosis for water purification.

## 7. Conclusions

As the core of the technology, forward osmosis (FO) membranes directly determine the separation efficiency and system stability. At present, the mainstream membrane materials include cellulose acetate (CA) membranes, thin-film composite (TFC) membranes, and aquaporin (AQP)-based biomimetic membranes.

As the driving force source of the FO process, draw solutions are divided into five categories: gaseous, organic compounds, inorganic compounds, magnetic nanoparticle-based, and polymer gel. An ideal draw solution needs to balance high osmotic pressure, low reverse diffusion, and easy recovery. At present, inorganic salt-based draw solutions are the most widely used, while new magnetic nanoparticle-based and polymer gel draw solutions provide new paths for solving the problems of reverse diffusion and energy consumption.

Membrane fouling is a key bottleneck restricting the large-scale application of FO technology, which is mainly divided into four types: inorganic fouling, organic fouling, colloidal fouling, and biofouling. Compared with pressure-driven membrane processes, FO technology has milder membrane fouling due to the absence of external pressure drive. However, the synergistic effect of different types of fouling during long-term operation will still lead to flux decline, which needs to be alleviated by combining membrane material modification and operation parameter optimization.

Forward osmosis technology has been effectively applied in many fields, such as seawater desalination, but it has great application potential in the energy field (shale gas development, mining development, lithium extraction from geothermal water) in the future. Especially in the field of lithium extraction from geothermal water, the energy synergy closed loop of “producing water with heat and extracting lithium with water” provides important support for the transformation of forward osmosis technology from water treatment to energy and resource recovery.

FO technology still has obvious deficiencies. Its water flux is generally lower than traditional RO technology; draw solution regeneration brings extra energy consumption; reverse solute flux is difficult to completely eliminate; and the supply of commercial FO membranes is limited. In the future, with the breakthrough in membrane material research and development, the improvement of new draw solution concentration methods, the deepening of applications in the energy field, and the expansion of application scenarios, FO technology is expected to realize the iteration from a “water treatment technology” to a “core technology for energy and resource recovery”, promote its industrial large-scale application, and provide important technical support for global water shortage and energy structure optimization.

Despite the promising prospects of FO technology, a series of key limitations still restrict its large-scale promotion. The reconcentration and regeneration of draw media bring extra energy consumption and operating costs. Reverse solute flow and internal concentration polarization remain universal problems that impair separation performance. Advanced FO membranes also face challenges in long-term operational stability when coping with complex water quality. Meanwhile, mixed fouling formed by the interaction of multiple foulants is hard to control completely. In addition, most relevant studies are still at laboratory or pilot stages, and systematic verification in full-scale industrial systems is insufficient. Future research needs to target these bottlenecks to further unlock the practical value of FO technology.

## Figures and Tables

**Table 1:** Comparison of Preparation Methods and Performances of Different Types of Representative Forward Osmosis Membranes [20,32,33,34,35,36,37].

Membrane Materials	Preparation Methods	Feed Solution	Draw Solution	Orientation	Water Flux (L·m^−2^·h^−1^)	Reverse Salt Diffusion Flux (g·m^−2^·h^−1^)	References
Cellulose triacetate (CTA) membranes	Phase Inversion	10 mmol/L NaCl	0.5 mol/L NaCl	AL-FS	4.42~9.03	0.6~5.3	[20]
Thin-film composite (TFC) membranes	Phase Inversion + Interfacial Polymerization	10 mmol/L NaCl	0.5 mol/L NaCl	AL-FS	9.5~12.0	2.4~4.9	[32]
Thin-film composite (TFC) membranes	Phase Inversion + Interfacial Polymerization	Deionized Water	1.5 mol/L NaCl	AL-DS	18.16	/	[33]
Aquaporin (AQP) membranes	Vesicle Rupture Method	200 mg/L NaCl	0.3 mol/L C_12_H_22_O_11_	AL-DS	16.4	6.6	[34]
Aquaporin (AQP) membranes	Encapsulate vesicles by forming a multilayer coating through polydopamine-histidine	Deionized Water	0.5 mol/L NaCl	AL-DS	43.6	8.9	[35]
Aquaporin (AQP) membranes	Encapsulate AQP-containing vesicles by combining magnetic assistance with the Layer-by-Layer (LbL) method	200 mg/L MgCl_2_	0.3 mol/L C_12_H_22_O_11_	AL-FS	21.8	2.4	[36,37]

Note: AL-FS: Active Layer facing Feed Solution. AL-DS: Active Layer facing Draw Solution. All experiments were conducted at 20–25 °C. Membrane orientation (AL-FS/AL-DS), feed solution and draw solution are specified for each group.

**Table 2:** Comprehensive performance comparison of typical FO membranes [32,33,34,35,36,37,38].

Membrane Type	Water Flux	Reverse Salt Flux	Salt Rejection (%)	Mechanical Strength	pH Stability	Chlorine Tolerance	Relative Cost	Key Trade-Offs & Characteristics	References
	(L⋅m^−2^⋅h^−1^)	(g⋅m^−2^⋅h^−1^)							
Cellulose Acetate (CA/CTA)	4.42–9.03	0.6–5.3	90–97	Moderate	Narrow (pH 4–7)	Excellent	Low	Pros: Low cost, easy fabrication, outstanding chlorine resistance, mild fouling tendency	[32,33]
								Cons: Prone to hydrolysis, poor alkali/acid resistance, limited flux improvement	
Thin-Film Composite (TFC)	9.5–18.16	2.4–4.9	95–99	High	Wide (pH 2–11)	Poor	Medium	Pros: Ultrahigh water flux, excellent salt rejection, tunable structure, strong mechanical stability	[33,34]
								Cons: Highly sensitive to free chlorine, vulnerable to organic and biofouling	
Aquaporin (AQP) Biomimetic	16.4–43.6	2.4–8.9	96–99.5	Low–Moderate	Medium (pH 3–9)	Medium	Very High	Pros: Extreme water permeability, superior selective separation performance	[35,36,37,38]
								Cons: Expensive raw materials, fragile structure, AQP activity easily deactivated, high reverse salt flux in partial formulations	

**Table 3:** Comparison of different draw solution categories in forward osmosis.

Draw Solution Type	Osmotic Pressure	Reverse Flux	Recovery Method	Energy Consumption	Practical Limitations	References
Gaseous	Very High	Moderate	Thermal	Moderate to High	Toxicity, corrosion	[44,45]
Organic	Moderate	Low	Thermal/Membrane	Moderate	Low flux, high cost	[48,55]
Inorganic	High	High	RO/NF	Low	Severe reverse diffusion	[46,56]
Magnetic Nanoparticles	Moderate to High	Low	Magnetic field	Low	Agglomeration, severe ICP	[7,62]
Polymer Gel	Low to Moderate	Very Low	Heating/Extrusion	Low	Slow water release, low flux	[66,68]

**Table 4:** Manifestations and Elimination Methods of Different Types of Forward Osmosis Membrane Fouling [69,70,71,72,73].

Membrane Fouling Types	Fouling Substances	Manifestations	Cleaning Measures and Effects	References
Inorganic fouling	Slightly soluble salts such as CaSO_4_, CaCO_3_, BaSO_4_	Scaling	After 15 min of cross-flow flushing at 22 cm/s, the water flux recovered by 96%	[69]
Colloidal fouling	Silicon nanoparticles, iron oxides, aluminum oxides, etc.	Filter cake layer	After 1 h of cross-flow flushing at 34.44 cm/s, the water flux was completely recovered	[70]
Organic fouling	Alginates, humic acid, proteins, etc.	Filter cake layer	After 15 min of cross-flow flushing at 60 L/h, the water flux recovered by 96%	[71]
	Actual oily wastewater		After 30 min of osmotic backwashing at 16 cm/s, the water flux recovered by 95%	[72]
Biofouling	Microorganisms, EPS (Extracellular Polymeric Substances)	Biofilm	After 1 h of cross-flow flushing at 33 cm/s combined with 100 mg/L NaClO, the water flux was completely recovered	[73]

## Data Availability

The original contributions presented in the study are included in the article, further inquiries can be directed to the corresponding author.
